# Incorporation of μ_3_-CO_3_ into an Mn^III^/Mn^IV^ Mn_12_ cluster: {[(cyclam)Mn^IV^(μ-O)_2_Mn^III^(H_2_O)(μ-OH)]_6_(μ_3_-CO_3_)_2_}Cl_8_·24H_2_O

**DOI:** 10.1107/S1600536810034999

**Published:** 2010-09-08

**Authors:** Ben B. Levaton, Marilyn M. Olmstead

**Affiliations:** a Department of Chemistry, University of California, Davis, CA 95656, USA

## Abstract

The centrosymmetric title cluster, hexa­aquadi-μ_3_-carbonato-hexa­cyclamhexa-μ_2_-hydroxido-dodeca-μ_2_-oxido-hexa­mang­an­ese(IV)hexa­manganese(III) octa­chloride tetra­cosa­hydrate, [Mn_12_(CO_3_)_2_O_12_(OH)_6_(C_10_H_24_N_4_)_6_(H_2_O)_6_]Cl_8_·24H_2_O, has two μ_3_-CO_3_ groups that not only bridge octahedrally coordinated Mn^III^ ions but also act as acceptors to two different kinds of hydrogen bonds. The carbonate anion is planar within experimental error and has an average C—O distance of 1.294 (4) Å. The crystal packing is stabilized by O—H⋯Cl, O—H⋯O, N—H⋯Cl and N—H⋯O hydrogen bonds. Two of the four independent chloride ions are disordered over five positions, and eight of the 12 independent water mol­ecules are disordered over 21 positions.

## Related literature

For the structure of an Mn_9_ cluster containing (μ_3_-CO_3_), see: Chakov *et al.* (2005 ▶). For some structures of Mn_12_ clusters containing Mn^III^/Mn^IV^, see: Lis (1980 ▶); Aubin *et al.* (1996 ▶); Sun *et al.* (1998 ▶); Kuroda-Sowa *et al.* (2001 ▶); Bian *et al.* (2004 ▶). For a recent structure of an Ag_17_ cluster that has incorporated atmospheric CO_2_ to encapsulate a carbonate, see: Bian *et al.* (2009 ▶). For bond-valence sum analysis for Mn—O, see: Palenik (1997 ▶).
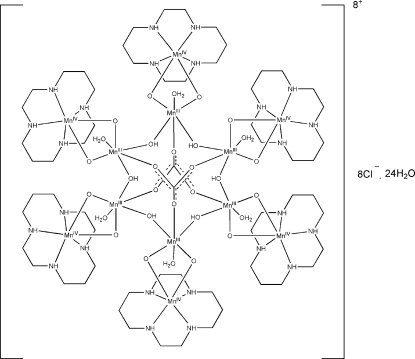


## Experimental

### 

#### Crystal data

[Mn_12_(CO_3_)_2_O_12_(OH)_6_(C_10_H_24_N_4_)_6_(H_2_O)_6_]Cl_8_·24H_2_O
*M* *_r_* = 3099.42
Triclinic, 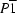

*a* = 15.2421 (15) Å
*b* = 15.5037 (15) Å
*c* = 17.1306 (17) Å
α = 90.707 (6)°
β = 114.523 (7)°
γ = 115.128 (7)°
*V* = 3245.8 (6) Å^3^
*Z* = 1
Mo *K*α radiation
μ = 1.38 mm^−1^
*T* = 90 K
0.43 × 0.18 × 0.14 mm

#### Data collection

Bruker SMART APEXII diffractometer
Absorption correction: multi-scan (*SADABS*; Sheldrick, 1996 ▶) *T*_min_ = 0.589, *T*_max_ = 0.831
40135 measured reflections
14856 independent reflections
10648 reflections with *I* > 2σ(*I*)
*R*_int_ = 0.042

#### Refinement

*R*[*F* ^2^ > 2σ(*F* ^2^)] = 0.051
*wR*(*F* ^2^) = 0.167
*S* = 1.14
14856 reflections
787 parameters
12 restraints
H atoms treated by a mixture of independent and constrained refinement
Δρ_max_ = 1.40 e Å^−3^
Δρ_min_ = −1.14 e Å^−3^

### 

Data collection: *APEX2* (Bruker, 2009 ▶); cell refinement: *SAINT* (Bruker, 2009 ▶); data reduction: *SAINT*; program(s) used to solve structure: *SHELXS97* (Sheldrick, 2008 ▶); program(s) used to refine structure: *SHELXL97* (Sheldrick, 2008 ▶); molecular graphics: *SHELXTL* (Sheldrick, 2008 ▶); software used to prepare material for publication: *SHELXL97*.

## Supplementary Material

Crystal structure: contains datablocks I, global. DOI: 10.1107/S1600536810034999/ci5168sup1.cif

Structure factors: contains datablocks I. DOI: 10.1107/S1600536810034999/ci5168Isup2.hkl

Additional supplementary materials:  crystallographic information; 3D view; checkCIF report

## Figures and Tables

**Table:** 

Mn1—O2	1.779 (3)
Mn1—O1	1.792 (2)
Mn1—N3	2.037 (4)
Mn1—N1	2.041 (4)
Mn1—N2	2.100 (3)
Mn1—N4	2.100 (3)
Mn2—O1	1.867 (3)
Mn2—O2	1.892 (3)
Mn2—O5	1.937 (2)
Mn2—O6	1.943 (2)
Mn2—O3	2.327 (3)
Mn2—O4	2.339 (3)
Mn3—O8	1.783 (2)
Mn3—O7	1.797 (2)
Mn3—N5	2.043 (3)
Mn3—N7	2.047 (3)
Mn3—N8	2.098 (3)
Mn3—N6	2.105 (3)
Mn4—O8	1.879 (2)
Mn4—O7	1.883 (2)
Mn4—O11	1.935 (2)
Mn4—O5^i^	1.943 (3)
Mn4—O9	2.311 (3)
Mn4—O10	2.315 (2)
Mn5—O15	1.783 (3)
Mn5—O14	1.788 (3)
Mn5—N11	2.034 (4)
Mn5—N9	2.038 (3)
Mn5—N10	2.093 (3)
Mn5—N12	2.095 (4)
Mn6—O14	1.868 (3)
Mn6—O15	1.883 (2)
Mn6—O11	1.937 (3)
Mn6—O6	1.949 (2)
Mn6—O13	2.302 (2)
Mn6—O12	2.335 (3)
O3—C31	1.293 (4)
C31—O13^i^	1.289 (4)
C31—O10	1.301 (4)

**Table:** 

O13^i^—C31—O3	120.8 (3)
O13^i^—C31—O10	120.0 (3)
O3—C31—O10	119.2 (3)

Symmetry code: (i) 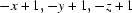.

**Table 2:** Hydrogen-bond geometry (Å, °)

*D*—H⋯*A*	*D*—H	H⋯*A*	*D*⋯*A*	*D*—H⋯*A*
N6—H6⋯Cl1	0.93	2.51	3.413 (3)	163
N8—H8⋯Cl1	0.93	2.27	3.198 (3)	174
O4—H4*E*⋯Cl2	0.82 (4)	2.33 (3)	3.106 (3)	158 (4)
N1—H1⋯Cl2	0.93	2.49	3.285 (4)	143
N2—H2⋯Cl3	0.93	2.46	3.338 (4)	157
N4—H4⋯Cl3	0.93	2.46	3.384 (4)	174
N11—H11⋯Cl4	0.93	2.37	3.148 (5)	141
N10—H10⋯Cl5*A*	0.93	2.68	3.523 (6)	152
N10—H10⋯Cl5*B*	0.93	2.49	3.185 (12)	132
N5—H5⋯Cl6	0.93	2.32	3.138 (4)	147
O4—H4*D*⋯O14	0.82 (4)	1.90 (2)	2.701 (4)	166 (4)
O5—H5*D*⋯O13	0.84 (4)	1.86 (2)	2.674 (3)	164 (4)
O6—H6*D*⋯O10	0.82 (4)	1.88 (2)	2.681 (4)	166 (4)
O9—H9*E*⋯O1^i^	0.87 (3)	1.83 (2)	2.665 (4)	161 (4)
O11—H11*D*⋯O3^i^	0.83 (2)	1.85 (2)	2.675 (3)	172 (4)
O12—H12*C*⋯O7	0.83 (5)	1.88 (5)	2.704 (4)	171 (4)
O12—H12*D*⋯Cl4	0.82 (2)	2.26 (2)	3.065 (4)	165 (4)
N2—H2⋯O28	0.93	2.08	2.952 (9)	156
N3—H3⋯O3	0.93	1.99	2.798 (4)	144
N7—H7⋯O10	0.93	1.99	2.790 (4)	143
N9—H9⋯O13	0.93	2.14	2.896 (4)	138
N10—H10⋯O20	0.93	2.02	2.912 (10)	160

Symmetry code: (i) 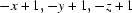.

**Fig. 1.**
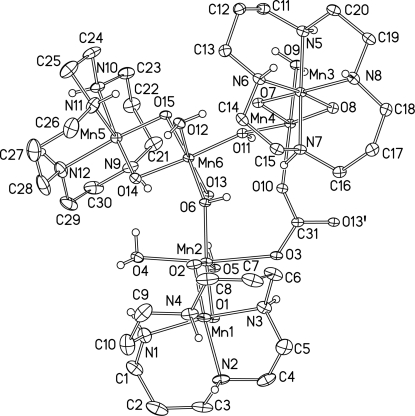
A drawing of the asymmetric unit of the title compound. Thermal ellipsoids are drawn at the 30% probability level. Hydrogen atoms bonded to carbon, chloride counterions, and hydrate molecules have been omitted for clarity. One of the oxygen atoms (O13') is shown at its symmetry position, ' = 1 - x, 1 - y, 1 - z, in order to show the complete carbonate anion.

**Fig. 2.**
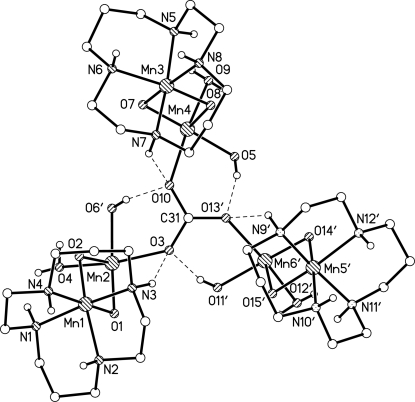
A view of one-half of the cluster normal to the triply bridging carbonate group. A portion of the hydrogen bonding is also depicted. Symmetry code: ' = 1 - x, 1 - y, 1 - z.

**Fig. 3.**
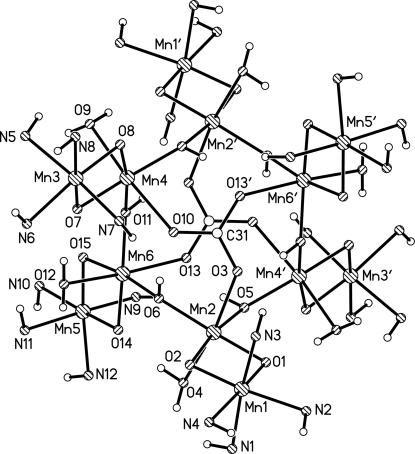
A view of the Mn12 cluster; CH2 groups have been omitted for clarity. Symmetry code: ' = 1 - x, 1 - y, 1 - z.

## supplementary crystallographic information

### Comment

Reactions of carbon dioxide with metals and metal clusters have recently attracted a great deal of interest (Bian *et al.*, 2009). Complexes of manganese rarely exhibit such reactivity. We wish to report the fortuitous fixation of carbon dioxide as carbonate ion in a mixed valent Mn^III^/Mn^IV^ cluster of 12 metal ions. An interesting structural feature consists of two triply bridged carbonate anions on opposite faces of the cluster. Previously, a Mn^III^ cluster containing carbonate was reported (Chakov *et al.*, 2005), but it has little in common with the title compound. Many Mn_12_ clusters are known, some of which have mixed valent Mn^III^/Mn^IV^ ions (Sun *et al.*, 1998; Kuroda-Sowa *et al.*, 2001; Bian *et al.*, 2004). The most famous is Mn_12_O_12_(MeCO_2_)_16_(H_2_O)_4_ (Lis, 1980; Aubin, *et al.*, 1996), which opened up the field of single molecule magnets. These structures are unlike that of the title compound. They contain an internal cubane of four Mn^IV^ ions with µ_3_-O bridges and eight outer Mn^III^ ions. The production of the title compound was unexpected. We presume that prolonged stirring of a manganese(II) chloride solution in an open vessel in the presence of base (cyclam, (1,4,8,11-tetraazacyclotetradecane)) yielded a basic Mn^III^/Mn^IV^ oxide which took up CO_2_ from the air and formed the triply bridged carbonate species of the title compound.

The cluster of the title compound has a center of symmetry. It has the overall formula {[(cyclam)Mn^IV^(µ-O)_2_Mn^III^(H_2_O)(µ-OH)]_6_(µ_3_-CO_3_)}_2_}^8+^ with charge balanced by chloride ions. There are 24 molecules of non-coordinated water in the model, many of which are disordered. The Mn_12_ cluster is shown in the Scheme.

For simplicity, only one half of the cluster is depicted in Fig. 1. The atoms labeled Mn1, Mn3, and Mn5 are Mn^IV^ and those labeled Mn2, Mn4, Mn6 are Mn^III^. The oxidation states for the Mn ions were verified by Bond Valence Sum analysis (Palenik, 1997). Details of the bond distances and angles are given in Table 1. The asymmetric unit of the title compound contains a carbonate anion coordinated to three Mn^III^ ions through each of its three O atoms. Within experimental error, the µ_3_-CO_3_ group is planar. The average C—O distance is 1.294 (4) Å. Each carbonate O atom accepts an intramolecular hydrogen bond from a µ-(OH) donor group that is ligated to two Mn^III^'s. A second hydrogen bond to each carbonate oxygen is formed by donation from a N—H group of cyclam, as shown in Figure 2. A single water molecule is coordinated to each Mn^III^ in a position *trans*- to the carbonate oxygen. The Mn—O(carbonate) distances, average 2.315 (8) Å, and Mn—O(H_2_O) distances, average 2.328 (11) Å, are long, indicating a Jahn-Teller effect of the d^4^ ion and strong *trans*-effect of carbonate. The remainder of the coordination sphere of the Mn^III^ ion is made up of two oxo bridges, average distance 1.879 (7) Å. These bridges link the Mn^III^ ions to Mn^IV^ ions. The average Mn^IV^—O distance is 1.787 (6) Å. The coordination sphere of the Mn^IV^ ions is completed by four amino N atoms of the cyclam ligand. The Mn—N bond distances reflect the *trans*-effect of the µ-oxo bridges; the average Mn^IV^—N(*trans*) distance is 2.097 (5)Å as compared to 2.040 (4)Å for Mn^IV^—N(*cis*). Fig. 3 depicts the entire Mn_12_ cluster with cyclam CH_2_ groups omitted for clarity. In sum, both Mn^III^ and Mn^IV^ have coordination number six and a pseudo- octahedral geometry. The inversion-related halves of the cluster are connected *via* the µ-(OH) groups. A diverse set of Mn—O bonds is exhibited in the structure, involving oxo, hydroxo, and aqua ligation to Mn^III^ and Mn^IV^ ions as well as intramolecular hydrogen bonding. The chloride counterions are primarily nestled in cyclam cavities, hydrogen bonded to N—H donor groups of the cylam ligands as well as to non-coordinated water molecules.

### Experimental

To a mixture of MnCl_2_.4H_2_O (136 mg, 687 mmol), cyclam (1,4,8,11-tetraazacyclotetradecane) (144 mg, 722 mmol), and sodium tetraphenylborate (289 mg, 844 mmol) in a 200 ml round bottom flask was added 150 ml of acetonitrile. The reaction was continuously stirred for 5 days over which time it turned from pale yellow to dark brown to dark olive green and a solid material was formed. The solid was filtered and redissolved in a 1:2 mixture of H_2_O:acetonitrile and placed in upcapped 5 mm diameter tubes in the refrigerator. After 2 weeks, black plates formed. The crystal selected for data collection was cut from a large plate.

### Refinement

Hydrogen atoms on water O and aza-N atoms were located in a difference map and subsequently refined with *U*_iso_ = 1.2*U*_eq_(N or O) and distance restraints of 0.84 (1) Å for O—H, 0.93 Å for N—H and H···H of 1.32 (3) Å for water. The C—H geometry was determined by idealized geometry and a C—H distance of 0.99 Å. The C—H and N—H H atoms were refined as riding on the parent atoms. There are seven different positions for the four chloride ions in the asymmetric unit. Of these, Cl1 and Cl2 are included at full occupancy while Cl3, Cl4, and Cl6 are at half occupancy and Cl5A/Cl5B respresent a split position of occupancy 0.40/0.10 occupancy. These disordered chlorides were selected based on longer hydrogen bonding distances and reasonable distribution within the structure. Four hydrate water O atoms, O16, O17, O18, and O19, were in sites of full occupancy and were refined with anisotropic thermal parameters. The remainder were refined with isotropic thermal parameters and fixed occupancies that were determined by an *ad hoc* method. Most of the hydrogen atoms were not reliably located for the hydrate molecules and none were included in the structure factor calculation. The final difference map contains a number of peaks in the region of the chloride ions and solvate water molecules that are possibly additional minor water sites or part of disordered chloride sites.

### Crystal data

**Table:**

[Mn_12_(CO_3_)_2_O_12_(OH)_6_(C_10_H_24_N_4_)_6_(H_2_O)_6_]Cl_8_·24H_2_O	*Z* = 1
*M**_r_* = 3099.42	*F*(000) = 1618
Triclinic, *P*1	*D*_x_ = 1.586 Mg m^−^^3^
Hall symbol: -P 1	Mo *K*α radiation, λ = 0.71073 Å
*a* = 15.2421 (15) Å	Cell parameters from 9868 reflections
*b* = 15.5037 (15) Å	θ = 2.6–29.4°
*c* = 17.1306 (17) Å	µ = 1.38 mm^−^^1^
α = 90.707 (6)°	*T* = 90 K
β = 114.523 (7)°	Parallelepiped, black
γ = 115.128 (7)°	0.43 × 0.18 × 0.14 mm
*V* = 3245.8 (6) Å^3^	

### Data collection

**Table:**

Bruker SMART APEXII diffractometer	14856 independent reflections
Radiation source: fine-focus sealed tube	10648 reflections with *I* > 2σ(*I*)
graphite	*R*_int_ = 0.042
Detector resolution: 8.3 pixels mm^-1^	θ_max_ = 27.5°, θ_min_ = 2.7°
ω scans	*h* = −19→19
Absorption correction: multi-scan (*SADABS*; Sheldrick, 1996)	*k* = −20→20
*T*_min_ = 0.589, *T*_max_ = 0.831	*l* = −22→22
40135 measured reflections	

### Refinement

**Table:**

Refinement on *F*^2^	Primary atom site location: structure-invariant direct methods
Least-squares matrix: full	Secondary atom site location: difference Fourier map
*R*[*F*^2^ > 2σ(*F*^2^)] = 0.051	Hydrogen site location: difference Fourier map
*wR*(*F*^2^) = 0.167	H atoms treated by a mixture of independent and constrained refinement
*S* = 1.14	*w* = 1/[σ^2^(*F*_o_^2^) + (0.0959*P*)^2^] where *P* = (*F*_o_^2^ + 2*F*_c_^2^)/3
14856 reflections	(Δ/σ)_max_ = 0.006
787 parameters	Δρ_max_ = 1.40 e Å^−^^3^
12 restraints	Δρ_min_ = −1.14 e Å^−^^3^

### Special details

**Table:**

Geometry. All e.s.d.'s (except the e.s.d. in the dihedral angle between two l.s. planes) are estimated using the full covariance matrix. The cell e.s.d.'s are taken into account individually in the estimation of e.s.d.'s in distances, angles and torsion angles; correlations between e.s.d.'s in cell parameters are only used when they are defined by crystal symmetry. An approximate (isotropic) treatment of cell e.s.d.'s is used for estimating e.s.d.'s involving l.s. planes.
Refinement. Refinement of *F*^2^ against ALL reflections. The weighted *R*-factor *wR* and goodness of fit *S* are based on *F*^2^, conventional *R*-factors *R* are based on *F*, with *F* set to zero for negative *F*^2^. The threshold expression of *F*^2^ > σ(*F*^2^) is used only for calculating *R*-factors(gt) *etc*. and is not relevant to the choice of reflections for refinement. *R*-factors based on *F*^2^ are statistically about twice as large as those based on *F*, and *R*- factors based on ALL data will be even larger.

### Fractional atomic coordinates and isotropic or equivalent isotropic displacement parameters (Å^2^)

**Table:**

	*x*	*y*	*z*	*U*_iso_*/*U*_eq_	Occ. (<1)
Mn1	0.61048 (5)	0.82243 (4)	0.74029 (4)	0.02005 (14)	
Mn2	0.48190 (5)	0.66143 (4)	0.60353 (4)	0.01677 (13)	
Mn3	0.68442 (4)	0.74479 (4)	0.31273 (4)	0.01583 (13)	
Mn4	0.56057 (4)	0.57784 (4)	0.34706 (3)	0.01455 (13)	
Mn5	0.10236 (4)	0.49328 (4)	0.28193 (4)	0.01948 (14)	
Mn6	0.32112 (4)	0.55349 (4)	0.36584 (3)	0.01522 (13)	
O1	0.5427 (2)	0.69222 (18)	0.72622 (16)	0.0195 (5)	
O2	0.5479 (2)	0.79948 (18)	0.62323 (17)	0.0235 (6)	
O3	0.6380 (2)	0.65305 (17)	0.62194 (16)	0.0174 (5)	
C31	0.6579 (3)	0.6311 (2)	0.5602 (2)	0.0160 (7)	
O4	0.3075 (2)	0.6456 (2)	0.55926 (19)	0.0259 (6)	
H4D	0.279 (3)	0.631 (3)	0.5055 (13)	0.031*	
H4E	0.310 (4)	0.696 (2)	0.578 (3)	0.031*	
O5	0.4100 (2)	0.52276 (17)	0.59896 (16)	0.0156 (5)	
H5D	0.365 (3)	0.495 (3)	0.5456 (14)	0.019*	
O6	0.4395 (2)	0.64189 (17)	0.47837 (16)	0.0157 (5)	
H6D	0.500 (2)	0.658 (3)	0.481 (3)	0.019*	
O7	0.5504 (2)	0.68689 (17)	0.30572 (16)	0.0171 (5)	
O8	0.6949 (2)	0.63902 (17)	0.34421 (17)	0.0180 (5)	
O9	0.4763 (2)	0.46920 (19)	0.21367 (17)	0.0217 (6)	
H9D	0.433 (3)	0.457 (3)	0.1600 (14)	0.026*	
H9E	0.457 (3)	0.4095 (17)	0.221 (3)	0.026*	
O10	0.62797 (19)	0.66251 (17)	0.48852 (16)	0.0161 (5)	
O11	0.41448 (19)	0.52118 (17)	0.33573 (16)	0.0153 (5)	
H11D	0.395 (3)	0.4645 (17)	0.344 (3)	0.018*	
O12	0.3795 (2)	0.6940 (2)	0.31251 (18)	0.0225 (6)	
H12C	0.431 (3)	0.695 (3)	0.306 (3)	0.027*	
H12D	0.337 (3)	0.700 (3)	0.2668 (19)	0.027*	
O13	0.2941 (2)	0.42210 (17)	0.43119 (16)	0.0168 (5)	
O14	0.2164 (2)	0.56917 (18)	0.38531 (17)	0.0207 (6)	
O15	0.1984 (2)	0.47938 (18)	0.25634 (16)	0.0186 (5)	
N1	0.4899 (3)	0.8385 (2)	0.7517 (3)	0.0329 (9)	
H1	0.4326	0.8173	0.6946	0.039*	
N2	0.6871 (3)	0.8367 (2)	0.8772 (2)	0.0299 (8)	
H2	0.7216	0.9024	0.9049	0.036*	
N3	0.7501 (3)	0.8279 (2)	0.7488 (2)	0.0277 (8)	
H3	0.7309	0.7636	0.7277	0.033*	
N4	0.6771 (3)	0.9739 (2)	0.7492 (2)	0.0308 (8)	
H4	0.7301	1.0060	0.8071	0.037*	
N5	0.6293 (3)	0.6958 (2)	0.1816 (2)	0.0220 (7)	
H5	0.6090	0.6294	0.1742	0.026*	
N6	0.6648 (3)	0.8692 (2)	0.2847 (2)	0.0198 (7)	
H6	0.7222	0.9115	0.2747	0.024*	
N7	0.7583 (3)	0.8187 (2)	0.4409 (2)	0.0197 (7)	
H7	0.7066	0.7878	0.4604	0.024*	
N8	0.8384 (3)	0.7991 (2)	0.3170 (2)	0.0222 (7)	
H8	0.8620	0.8644	0.3140	0.027*	
N9	0.0652 (3)	0.3703 (2)	0.3302 (2)	0.0280 (8)	
H9	0.1263	0.3867	0.3841	0.034*	
N10	−0.0219 (3)	0.4080 (3)	0.1564 (2)	0.0276 (8)	
H10	−0.0890	0.3919	0.1544	0.033*	
N11	0.1099 (3)	0.6064 (3)	0.2213 (3)	0.0349 (9)	
H11	0.1700	0.6246	0.2111	0.042*	
N12	−0.0018 (3)	0.5104 (3)	0.3234 (3)	0.0381 (9)	
H12	−0.0707	0.4860	0.2751	0.046*	
C1	0.4406 (4)	0.7784 (4)	0.8054 (3)	0.0433 (12)	
H1A	0.4079	0.7080	0.7793	0.052*	
H1B	0.3815	0.7913	0.8033	0.052*	
C2	0.5257 (5)	0.8021 (4)	0.9006 (4)	0.0575 (16)	
H2A	0.5589	0.8729	0.9252	0.069*	
H2B	0.4879	0.7665	0.9342	0.069*	
C3	0.6154 (4)	0.7779 (4)	0.9158 (3)	0.0444 (13)	
H3A	0.5829	0.7076	0.8898	0.053*	
H3B	0.6608	0.7894	0.9801	0.053*	
C4	0.7723 (4)	0.8054 (3)	0.8941 (3)	0.0413 (12)	
H4A	0.7376	0.7333	0.8752	0.050*	
H4B	0.8241	0.8252	0.9579	0.050*	
C5	0.8319 (4)	0.8533 (4)	0.8431 (3)	0.0423 (12)	
H5A	0.8724	0.9252	0.8659	0.051*	
H5B	0.8852	0.8299	0.8494	0.051*	
C6	0.7960 (4)	0.8854 (3)	0.6931 (3)	0.0396 (12)	
H6A	0.7425	0.8548	0.6302	0.048*	
H6B	0.8635	0.8823	0.7036	0.048*	
C7	0.8227 (4)	0.9928 (3)	0.7118 (3)	0.0455 (14)	
H7A	0.8752	1.0225	0.7750	0.055*	
H7B	0.8593	1.0275	0.6772	0.055*	
C8	0.7266 (5)	1.0086 (3)	0.6908 (3)	0.0465 (14)	
H8A	0.7496	1.0794	0.6956	0.056*	
H8B	0.6709	0.9741	0.6291	0.056*	
C9	0.5843 (4)	0.9973 (3)	0.7282 (4)	0.0476 (14)	
H9A	0.6131	1.0687	0.7463	0.057*	
H9B	0.5341	0.9757	0.6640	0.057*	
C10	0.5250 (4)	0.9442 (4)	0.7779 (4)	0.0535 (15)	
H10A	0.5739	0.9690	0.8421	0.064*	
H10B	0.4606	0.9543	0.7633	0.064*	
C11	0.5300 (3)	0.7012 (3)	0.1180 (3)	0.0270 (9)	
H11A	0.4682	0.6607	0.1297	0.032*	
H11B	0.5108	0.6727	0.0575	0.032*	
C12	0.5439 (3)	0.8035 (3)	0.1223 (3)	0.0275 (9)	
H12A	0.4782	0.8016	0.0737	0.033*	
H12B	0.6075	0.8445	0.1128	0.033*	
C13	0.5608 (3)	0.8510 (3)	0.2084 (3)	0.0244 (8)	
H13A	0.5596	0.9141	0.2030	0.029*	
H13B	0.4991	0.8084	0.2195	0.029*	
C14	0.6760 (3)	0.9175 (3)	0.3670 (3)	0.0234 (8)	
H14A	0.6088	0.8804	0.3730	0.028*	
H14B	0.6870	0.9849	0.3645	0.028*	
C15	0.7724 (3)	0.9199 (3)	0.4441 (3)	0.0248 (9)	
H15A	0.8407	0.9634	0.4417	0.030*	
H15B	0.7774	0.9457	0.4998	0.030*	
C16	0.8569 (3)	0.8134 (3)	0.5043 (3)	0.0246 (8)	
H16A	0.8366	0.7444	0.5077	0.030*	
H16B	0.8832	0.8512	0.5634	0.030*	
C17	0.9504 (3)	0.8531 (3)	0.4794 (3)	0.0266 (9)	
H17A	0.9682	0.9212	0.4734	0.032*	
H17B	1.0160	0.8550	0.5280	0.032*	
C18	0.9251 (3)	0.7943 (3)	0.3954 (3)	0.0247 (8)	
H18A	0.9924	0.8187	0.3886	0.030*	
H18B	0.9020	0.7251	0.3994	0.030*	
C19	0.8205 (3)	0.7441 (3)	0.2352 (3)	0.0272 (9)	
H19A	0.8085	0.6770	0.2407	0.033*	
H19B	0.8856	0.7774	0.2255	0.033*	
C20	0.7220 (3)	0.7402 (3)	0.1592 (3)	0.0296 (9)	
H20A	0.7044	0.6997	0.1044	0.035*	
H20B	0.7360	0.8068	0.1503	0.035*	
C21	0.0490 (4)	0.2806 (3)	0.2803 (3)	0.0384 (11)	
H21A	0.0326	0.2275	0.3118	0.046*	
H21B	0.1180	0.2943	0.2790	0.046*	
C22	−0.0434 (4)	0.2451 (3)	0.1854 (3)	0.0376 (11)	
H22A	−0.0541	0.1822	0.1587	0.045*	
H22B	−0.1121	0.2327	0.1867	0.045*	
C23	−0.0229 (4)	0.3161 (3)	0.1279 (3)	0.0334 (10)	
H23A	0.0483	0.3325	0.1298	0.040*	
H23B	−0.0806	0.2846	0.0662	0.040*	
C24	−0.0075 (4)	0.4717 (4)	0.0938 (3)	0.0409 (12)	
H24A	−0.0740	0.4424	0.0360	0.049*	
H24B	0.0553	0.4785	0.0852	0.049*	
C25	0.0127 (4)	0.5694 (4)	0.1313 (4)	0.0518 (14)	
H25A	−0.0522	0.5636	0.1357	0.062*	
H25B	0.0279	0.6151	0.0932	0.062*	
C26	0.1297 (4)	0.6964 (4)	0.2709 (4)	0.0506 (14)	
H26A	0.1339	0.7455	0.2343	0.061*	
H26B	0.2013	0.7233	0.3245	0.061*	
C27	0.0424 (5)	0.6807 (4)	0.2976 (5)	0.0619 (17)	
H27A	−0.0293	0.6516	0.2439	0.074*	
H27B	0.0563	0.7453	0.3241	0.074*	
C28	0.0351 (4)	0.6150 (4)	0.3628 (4)	0.0562 (16)	
H28A	0.1078	0.6414	0.4151	0.067*	
H28B	−0.0169	0.6162	0.3826	0.067*	
C29	−0.0100 (4)	0.4490 (4)	0.3874 (3)	0.0488 (14)	
H29A	−0.0731	0.4392	0.3969	0.059*	
H29B	0.0573	0.4817	0.4446	0.059*	
C30	−0.0244 (4)	0.3517 (4)	0.3525 (3)	0.0454 (13)	
H30A	−0.0218	0.3122	0.3976	0.055*	
H30B	−0.0962	0.3151	0.2994	0.055*	
Cl1	0.91039 (8)	1.01855 (7)	0.29167 (7)	0.0268 (2)	
Cl2	0.24589 (13)	0.79935 (12)	0.60522 (13)	0.0769 (6)	
Cl3	0.86737 (18)	1.07361 (15)	0.96304 (14)	0.0367 (5)	0.50
Cl4	0.2516 (2)	0.7178 (3)	0.1287 (2)	0.0673 (10)	0.50
Cl5A	−0.2407 (3)	0.4394 (3)	0.1490 (3)	0.0730 (12)	0.40
Cl5B	−0.2790 (9)	0.2686 (11)	0.0485 (8)	0.045 (3)	0.10
Cl6	0.5772 (2)	0.49181 (16)	0.08903 (17)	0.0465 (6)	0.50
O16	0.2661 (3)	0.9080 (3)	0.7683 (3)	0.0518 (10)	
O17	1.0355 (3)	1.1982 (3)	0.8871 (2)	0.0498 (9)	
O18	−0.2086 (3)	0.6154 (3)	0.0340 (2)	0.0507 (9)	
O19	0.8974 (3)	0.9445 (3)	0.1130 (3)	0.0585 (10)	
O20	−0.2526 (7)	0.3240 (7)	0.1109 (6)	0.068 (2)*	0.50
O21A	0.2119 (5)	0.4846 (5)	0.6239 (4)	0.0420 (15)*	0.55
O21B	0.1753 (6)	0.4493 (6)	0.5731 (5)	0.0388 (17)*	0.45
O22A	0.4103 (9)	0.9540 (8)	0.9533 (7)	0.061 (3)*	0.45
O22B	0.3601 (10)	0.9330 (9)	0.9500 (8)	0.049 (3)*	0.35
O22C	0.3772 (14)	0.8915 (13)	0.9507 (11)	0.037 (4)*	0.20
O23A	0.2723 (4)	0.4768 (4)	0.1348 (4)	0.0357 (12)*	0.60
O23B	0.2584 (7)	0.4259 (7)	0.1386 (5)	0.0369 (19)*	0.40
O24A	0.4901 (5)	0.8550 (4)	0.4605 (4)	0.0360 (13)*	0.60
O24B	0.4420 (7)	0.8431 (6)	0.4547 (5)	0.0337 (19)*	0.40
O25A	0.6386 (9)	1.0576 (7)	0.4969 (6)	0.037 (2)*	0.35
O25B	0.5869 (11)	1.0364 (9)	0.4906 (8)	0.030 (3)*	0.25
O25C	0.7427 (14)	1.1185 (12)	0.5456 (11)	0.038 (4)*	0.20
O25D	0.7994 (10)	1.1247 (9)	0.5422 (8)	0.017 (3)*	0.20
O26A	0.3055 (10)	0.8377 (9)	0.0711 (8)	0.045 (3)*	0.31
O26B	0.3085 (13)	0.8749 (13)	0.0392 (11)	0.075 (5)*	0.30
O27	0.8491 (6)	1.1410 (5)	0.9027 (5)	0.0325 (16)*	0.42
O28	0.7746 (6)	1.0212 (6)	1.0010 (5)	0.0361 (18)*	0.40
O29	0.2422 (9)	0.6563 (8)	0.1076 (7)	0.049 (3)*	0.40
O30	0.2682 (14)	0.6482 (13)	0.0730 (12)	0.091 (5)*	0.33
O31	0.7693 (9)	0.4686 (9)	0.2254 (8)	0.054 (3)*	0.34

### Atomic displacement parameters (Å^2^)

**Table:**

	*U*^11^	*U*^22^	*U*^33^	*U*^12^	*U*^13^	*U*^23^
Mn1	0.0274 (3)	0.0092 (3)	0.0139 (3)	0.0021 (2)	0.0082 (2)	−0.0014 (2)
Mn2	0.0219 (3)	0.0074 (2)	0.0125 (3)	0.0006 (2)	0.0073 (2)	−0.0006 (2)
Mn3	0.0165 (3)	0.0086 (3)	0.0168 (3)	0.0003 (2)	0.0087 (2)	0.0013 (2)
Mn4	0.0145 (3)	0.0078 (2)	0.0147 (3)	−0.0003 (2)	0.0068 (2)	0.0009 (2)
Mn5	0.0152 (3)	0.0159 (3)	0.0197 (3)	0.0015 (2)	0.0077 (2)	0.0024 (2)
Mn6	0.0143 (3)	0.0104 (3)	0.0128 (3)	0.0001 (2)	0.0053 (2)	−0.0018 (2)
O1	0.0248 (14)	0.0118 (12)	0.0140 (13)	0.0025 (10)	0.0086 (11)	0.0008 (10)
O2	0.0320 (15)	0.0104 (12)	0.0139 (13)	0.0036 (11)	0.0052 (12)	−0.0005 (10)
O3	0.0203 (13)	0.0113 (12)	0.0119 (12)	0.0011 (10)	0.0067 (10)	−0.0018 (9)
C31	0.0131 (16)	0.0070 (15)	0.0147 (18)	−0.0051 (13)	0.0053 (14)	−0.0015 (13)
O4	0.0355 (16)	0.0237 (15)	0.0235 (15)	0.0149 (13)	0.0171 (14)	0.0052 (12)
O5	0.0163 (12)	0.0103 (12)	0.0109 (12)	0.0005 (10)	0.0043 (10)	−0.0005 (9)
O6	0.0150 (12)	0.0112 (12)	0.0121 (12)	−0.0002 (10)	0.0054 (10)	−0.0021 (9)
O7	0.0181 (12)	0.0107 (12)	0.0167 (13)	0.0028 (10)	0.0073 (11)	0.0030 (10)
O8	0.0170 (12)	0.0098 (12)	0.0213 (14)	0.0015 (10)	0.0088 (11)	0.0026 (10)
O9	0.0269 (15)	0.0164 (13)	0.0145 (13)	0.0051 (11)	0.0088 (12)	0.0012 (11)
O10	0.0156 (12)	0.0107 (11)	0.0160 (13)	0.0008 (10)	0.0076 (10)	0.0015 (10)
O11	0.0162 (12)	0.0077 (11)	0.0160 (13)	0.0009 (10)	0.0072 (10)	0.0018 (10)
O12	0.0239 (15)	0.0227 (14)	0.0207 (14)	0.0105 (12)	0.0107 (12)	0.0062 (11)
O13	0.0172 (12)	0.0118 (12)	0.0137 (13)	0.0012 (10)	0.0065 (10)	0.0000 (10)
O14	0.0188 (13)	0.0166 (13)	0.0210 (14)	0.0036 (11)	0.0095 (11)	−0.0010 (11)
O15	0.0151 (12)	0.0180 (13)	0.0121 (12)	0.0002 (10)	0.0053 (10)	−0.0005 (10)
N1	0.033 (2)	0.0193 (18)	0.040 (2)	0.0093 (15)	0.0152 (18)	−0.0016 (16)
N2	0.037 (2)	0.0174 (17)	0.0154 (17)	0.0010 (15)	0.0069 (15)	−0.0011 (13)
N3	0.0266 (18)	0.0146 (16)	0.0287 (19)	−0.0007 (14)	0.0125 (15)	−0.0042 (14)
N4	0.041 (2)	0.0092 (15)	0.0228 (18)	0.0010 (14)	0.0093 (16)	−0.0027 (13)
N5	0.0236 (17)	0.0156 (15)	0.0230 (17)	0.0049 (13)	0.0122 (14)	0.0021 (13)
N6	0.0211 (16)	0.0117 (14)	0.0229 (17)	0.0015 (12)	0.0134 (14)	0.0027 (13)
N7	0.0196 (16)	0.0129 (15)	0.0174 (16)	−0.0001 (12)	0.0087 (13)	0.0007 (12)
N8	0.0222 (16)	0.0138 (15)	0.0291 (19)	0.0031 (13)	0.0161 (15)	0.0045 (13)
N9	0.0241 (17)	0.0188 (17)	0.0214 (18)	−0.0022 (14)	0.0065 (15)	0.0041 (14)
N10	0.0178 (16)	0.0301 (19)	0.0185 (17)	0.0007 (14)	0.0053 (14)	0.0044 (14)
N11	0.030 (2)	0.0240 (19)	0.040 (2)	0.0112 (16)	0.0080 (17)	0.0096 (17)
N12	0.0235 (19)	0.040 (2)	0.041 (2)	0.0077 (17)	0.0141 (18)	−0.0052 (18)
C1	0.050 (3)	0.038 (3)	0.045 (3)	0.016 (2)	0.030 (3)	0.002 (2)
C2	0.074 (4)	0.045 (3)	0.047 (3)	0.012 (3)	0.041 (3)	−0.005 (3)
C3	0.061 (3)	0.031 (3)	0.023 (2)	0.003 (2)	0.023 (2)	0.0019 (19)
C4	0.039 (3)	0.028 (2)	0.023 (2)	0.005 (2)	−0.003 (2)	−0.0016 (19)
C5	0.028 (2)	0.031 (3)	0.039 (3)	−0.001 (2)	0.007 (2)	−0.006 (2)
C6	0.042 (3)	0.025 (2)	0.037 (3)	−0.003 (2)	0.025 (2)	−0.0042 (19)
C7	0.052 (3)	0.020 (2)	0.039 (3)	−0.011 (2)	0.028 (3)	−0.0087 (19)
C8	0.068 (4)	0.016 (2)	0.027 (3)	0.002 (2)	0.017 (2)	0.0015 (18)
C9	0.049 (3)	0.020 (2)	0.047 (3)	0.014 (2)	0.002 (2)	0.001 (2)
C10	0.042 (3)	0.026 (3)	0.079 (4)	0.012 (2)	0.021 (3)	−0.010 (3)
C11	0.028 (2)	0.025 (2)	0.020 (2)	0.0079 (17)	0.0090 (18)	0.0011 (16)
C12	0.028 (2)	0.029 (2)	0.025 (2)	0.0144 (18)	0.0115 (18)	0.0074 (18)
C13	0.028 (2)	0.0187 (19)	0.027 (2)	0.0111 (17)	0.0125 (18)	0.0076 (16)
C14	0.029 (2)	0.0136 (18)	0.032 (2)	0.0077 (16)	0.0201 (18)	0.0043 (16)
C15	0.029 (2)	0.0116 (17)	0.026 (2)	0.0009 (16)	0.0152 (18)	−0.0032 (15)
C16	0.0218 (19)	0.0180 (19)	0.022 (2)	0.0020 (15)	0.0077 (16)	0.0012 (15)
C17	0.0167 (18)	0.0174 (19)	0.030 (2)	−0.0013 (15)	0.0075 (17)	0.0018 (16)
C18	0.0199 (19)	0.0191 (19)	0.031 (2)	0.0050 (16)	0.0123 (17)	0.0070 (17)
C19	0.034 (2)	0.025 (2)	0.030 (2)	0.0135 (18)	0.0213 (19)	0.0072 (17)
C20	0.034 (2)	0.030 (2)	0.026 (2)	0.0116 (19)	0.0189 (19)	0.0066 (18)
C21	0.036 (3)	0.016 (2)	0.034 (3)	−0.0011 (18)	0.005 (2)	0.0042 (18)
C22	0.028 (2)	0.021 (2)	0.035 (3)	−0.0041 (18)	0.006 (2)	−0.0037 (19)
C23	0.026 (2)	0.028 (2)	0.021 (2)	−0.0017 (18)	0.0049 (18)	−0.0031 (18)
C24	0.031 (2)	0.045 (3)	0.022 (2)	0.006 (2)	0.0033 (19)	0.016 (2)
C25	0.043 (3)	0.047 (3)	0.047 (3)	0.019 (3)	0.008 (3)	0.023 (3)
C26	0.045 (3)	0.026 (3)	0.069 (4)	0.017 (2)	0.016 (3)	0.008 (3)
C27	0.051 (3)	0.044 (3)	0.092 (5)	0.030 (3)	0.027 (3)	0.002 (3)
C28	0.037 (3)	0.052 (3)	0.075 (4)	0.017 (3)	0.027 (3)	−0.018 (3)
C29	0.034 (3)	0.061 (4)	0.041 (3)	0.004 (2)	0.028 (2)	−0.001 (3)
C30	0.036 (3)	0.043 (3)	0.033 (3)	−0.004 (2)	0.019 (2)	0.005 (2)
Cl1	0.0260 (5)	0.0144 (4)	0.0314 (5)	0.0009 (4)	0.0150 (4)	0.0040 (4)
Cl2	0.0516 (9)	0.0594 (10)	0.0962 (13)	0.0178 (8)	0.0241 (9)	−0.0287 (9)
Cl3	0.0369 (12)	0.0217 (10)	0.0243 (11)	−0.0075 (9)	0.0134 (9)	−0.0130 (8)
Cl4	0.0354 (14)	0.076 (2)	0.0549 (19)	0.0091 (15)	0.0080 (13)	0.0296 (17)
Cl5A	0.0283 (17)	0.079 (3)	0.078 (3)	0.0061 (17)	0.0153 (18)	0.008 (2)
Cl5B	0.020 (5)	0.065 (8)	0.041 (7)	0.017 (5)	0.009 (5)	0.016 (6)
Cl6	0.0596 (16)	0.0195 (10)	0.0430 (14)	0.0106 (10)	0.0176 (12)	−0.0006 (10)
O16	0.043 (2)	0.044 (2)	0.077 (3)	0.0195 (18)	0.037 (2)	0.028 (2)
O17	0.048 (2)	0.046 (2)	0.035 (2)	0.0090 (17)	0.0154 (17)	−0.0040 (16)
O18	0.049 (2)	0.049 (2)	0.038 (2)	0.0144 (18)	0.0151 (17)	−0.0024 (17)
O19	0.072 (3)	0.054 (2)	0.047 (2)	0.023 (2)	0.033 (2)	0.0101 (19)

### Geometric parameters (Å, °)

**Table:**

Mn1—O2	1.779 (3)	C1—C2	1.517 (8)
Mn1—O1	1.792 (2)	C1—H1A	0.99
Mn1—N3	2.037 (4)	C1—H1B	0.99
Mn1—N1	2.041 (4)	C2—C3	1.489 (8)
Mn1—N2	2.100 (3)	C2—H2A	0.99
Mn1—N4	2.100 (3)	C2—H2B	0.99
Mn1—Mn2	2.7295 (8)	C3—H3A	0.99
Mn2—O1	1.867 (3)	C3—H3B	0.99
Mn2—O2	1.892 (3)	C4—C5	1.497 (7)
Mn2—O5	1.937 (2)	C4—H4A	0.99
Mn2—O6	1.943 (2)	C4—H4B	0.99
Mn2—O3	2.327 (3)	C5—H5A	0.99
Mn2—O4	2.339 (3)	C5—H5B	0.99
Mn3—O8	1.783 (2)	C6—C7	1.531 (7)
Mn3—O7	1.797 (2)	C6—H6A	0.99
Mn3—N5	2.043 (3)	C6—H6B	0.99
Mn3—N7	2.047 (3)	C7—C8	1.484 (8)
Mn3—N8	2.098 (3)	C7—H7A	0.99
Mn3—N6	2.105 (3)	C7—H7B	0.99
Mn3—Mn4	2.7237 (8)	C8—H8A	0.99
Mn4—O8	1.879 (2)	C8—H8B	0.99
Mn4—O7	1.883 (2)	C9—C10	1.498 (8)
Mn4—O11	1.935 (2)	C9—H9A	0.99
Mn4—O5^i^	1.943 (3)	C9—H9B	0.99
Mn4—O9	2.311 (3)	C10—H10A	0.99
Mn4—O10	2.315 (2)	C10—H10B	0.99
Mn5—O15	1.783 (3)	C11—C12	1.505 (6)
Mn5—O14	1.788 (3)	C11—H11A	0.99
Mn5—N11	2.034 (4)	C11—H11B	0.99
Mn5—N9	2.038 (3)	C12—C13	1.510 (6)
Mn5—N10	2.093 (3)	C12—H12A	0.99
Mn5—N12	2.095 (4)	C12—H12B	0.99
Mn5—Mn6	2.7204 (9)	C13—H13A	0.99
Mn6—O14	1.868 (3)	C13—H13B	0.99
Mn6—O15	1.883 (2)	C14—C15	1.498 (6)
Mn6—O11	1.937 (3)	C14—H14A	0.99
Mn6—O6	1.949 (2)	C14—H14B	0.99
Mn6—O13	2.302 (2)	C15—H15A	0.99
Mn6—O12	2.335 (3)	C15—H15B	0.99
O3—C31	1.293 (4)	C16—C17	1.535 (5)
C31—O13^i^	1.289 (4)	C16—H16A	0.99
C31—O10	1.301 (4)	C16—H16B	0.99
O4—H4D	0.82 (4)	C17—C18	1.503 (6)
O4—H4E	0.82 (4)	C17—H17A	0.99
O5—Mn4^i^	1.943 (3)	C17—H17B	0.99
O5—H5D	0.84 (4)	C18—H18A	0.99
O6—H6D	0.82 (4)	C18—H18B	0.99
O9—H9D	0.84 (4)	C19—C20	1.500 (6)
O9—H9E	0.87 (3)	C19—H19A	0.99
O11—H11D	0.83 (4)	C19—H19B	0.99
O12—H12C	0.83 (5)	C20—H20A	0.99
O12—H12D	0.82 (5)	C20—H20B	0.99
O13—C31^i^	1.289 (4)	C21—C22	1.532 (6)
N1—C10	1.486 (6)	C21—H21A	0.99
N1—C1	1.509 (6)	C21—H21B	0.99
N1—H1	0.93	C22—C23	1.506 (6)
N2—C3	1.494 (6)	C22—H22A	0.99
N2—C4	1.495 (6)	C22—H22B	0.99
N2—H2	0.93	C23—H23A	0.99
N3—C5	1.489 (6)	C23—H23B	0.99
N3—C6	1.497 (5)	C24—C25	1.489 (8)
N3—H3	0.93	C24—H24A	0.99
N4—C8	1.468 (6)	C24—H24B	0.99
N4—C9	1.509 (6)	C25—H25A	0.99
N4—H4	0.93	C25—H25B	0.99
N5—C11	1.492 (5)	C26—C27	1.508 (8)
N5—C20	1.501 (5)	C26—H26A	0.99
N5—H5	0.93	C26—H26B	0.99
N6—C13	1.481 (5)	C27—C28	1.528 (9)
N6—C14	1.498 (5)	C27—H27A	0.99
N6—H6	0.93	C27—H27B	0.99
N7—C16	1.482 (5)	C28—H28A	0.99
N7—C15	1.488 (5)	C28—H28B	0.99
N7—H7	0.93	C29—C30	1.506 (8)
N8—C18	1.473 (5)	C29—H29A	0.99
N8—C19	1.490 (5)	C29—H29B	0.99
N8—H8	0.93	C30—H30A	0.99
N9—C30	1.483 (6)	C30—H30B	0.99
N9—C21	1.493 (6)	Cl3—O27	1.507 (8)
N9—H9	0.93	Cl3—O28	1.697 (8)
N10—C24	1.486 (5)	Cl4—O29	0.945 (10)
N10—C23	1.492 (6)	Cl4—O30	1.597 (19)
N10—H10	0.93	Cl4—O26A	2.127 (13)
N11—C26	1.470 (6)	Cl5A—O31^ii^	1.304 (12)
N11—C25	1.503 (6)	Cl5A—O20	1.806 (11)
N11—H11	0.93	Cl5B—O20	1.169 (16)
N12—C29	1.470 (7)	O29—O30	0.873 (17)
N12—C28	1.511 (6)	O31—Cl5A^iii^	1.304 (12)
N12—H12	0.93		
			
O2—Mn1—O1	86.28 (12)	C30—N9—H9	104.8
O2—Mn1—N3	92.60 (14)	C21—N9—H9	104.8
O1—Mn1—N3	94.14 (13)	Mn5—N9—H9	104.8
O2—Mn1—N1	95.52 (15)	C24—N10—C23	109.0 (4)
O1—Mn1—N1	93.32 (13)	C24—N10—Mn5	106.1 (3)
N3—Mn1—N1	169.31 (15)	C23—N10—Mn5	115.9 (3)
O2—Mn1—N2	173.95 (14)	C24—N10—H10	108.5
O1—Mn1—N2	89.08 (12)	C23—N10—H10	108.5
N3—Mn1—N2	83.87 (15)	Mn5—N10—H10	108.5
N1—Mn1—N2	88.61 (16)	C26—N11—C25	112.8 (4)
O2—Mn1—N4	90.63 (13)	C26—N11—Mn5	117.8 (3)
O1—Mn1—N4	175.16 (14)	C25—N11—Mn5	109.4 (3)
N3—Mn1—N4	89.71 (15)	C26—N11—H11	105.2
N1—Mn1—N4	83.26 (15)	C25—N11—H11	105.2
N2—Mn1—N4	94.25 (14)	Mn5—N11—H11	105.2
O2—Mn1—Mn2	43.57 (8)	C29—N12—C28	111.0 (4)
O1—Mn1—Mn2	42.83 (8)	C29—N12—Mn5	106.2 (3)
N3—Mn1—Mn2	92.08 (10)	C28—N12—Mn5	114.4 (3)
N1—Mn1—Mn2	98.60 (11)	C29—N12—H12	108.3
N2—Mn1—Mn2	131.44 (10)	C28—N12—H12	108.3
N4—Mn1—Mn2	134.20 (10)	Mn5—N12—H12	108.3
O1—Mn2—O2	81.01 (11)	N1—C1—C2	111.6 (4)
O1—Mn2—O5	91.51 (11)	N1—C1—H1A	109.3
O2—Mn2—O5	170.92 (11)	C2—C1—H1A	109.3
O1—Mn2—O6	171.82 (11)	N1—C1—H1B	109.3
O2—Mn2—O6	94.09 (11)	C2—C1—H1B	109.3
O5—Mn2—O6	93.92 (10)	H1A—C1—H1B	108.0
O1—Mn2—O3	87.64 (10)	C3—C2—C1	116.1 (4)
O2—Mn2—O3	94.66 (11)	C3—C2—H2A	108.3
O5—Mn2—O3	90.15 (10)	C1—C2—H2A	108.3
O6—Mn2—O3	86.24 (10)	C3—C2—H2B	108.3
O1—Mn2—O4	102.12 (11)	C1—C2—H2B	108.3
O2—Mn2—O4	92.91 (11)	H2A—C2—H2B	107.4
O5—Mn2—O4	83.57 (10)	C2—C3—N2	113.2 (4)
O6—Mn2—O4	84.58 (10)	C2—C3—H3A	108.9
O3—Mn2—O4	168.49 (9)	N2—C3—H3A	108.9
O1—Mn2—Mn1	40.72 (8)	C2—C3—H3B	108.9
O2—Mn2—Mn1	40.40 (8)	N2—C3—H3B	108.9
O5—Mn2—Mn1	132.21 (8)	H3A—C3—H3B	107.8
O6—Mn2—Mn1	133.67 (7)	N2—C4—C5	108.2 (4)
O3—Mn2—Mn1	89.21 (6)	N2—C4—H4A	110.1
O4—Mn2—Mn1	102.16 (8)	C5—C4—H4A	110.1
O8—Mn3—O7	86.63 (11)	N2—C4—H4B	110.1
O8—Mn3—N5	95.31 (12)	C5—C4—H4B	110.1
O7—Mn3—N5	94.42 (12)	H4A—C4—H4B	108.4
O8—Mn3—N7	92.66 (12)	N3—C5—C4	108.3 (4)
O7—Mn3—N7	93.53 (12)	N3—C5—H5A	110.0
N5—Mn3—N7	169.05 (12)	C4—C5—H5A	110.0
O8—Mn3—N8	88.84 (12)	N3—C5—H5B	110.0
O7—Mn3—N8	174.58 (12)	C4—C5—H5B	110.0
N5—Mn3—N8	83.04 (13)	H5A—C5—H5B	108.4
N7—Mn3—N8	89.64 (13)	N3—C6—C7	113.1 (4)
O8—Mn3—N6	173.89 (12)	N3—C6—H6A	109.0
O7—Mn3—N6	88.92 (12)	C7—C6—H6A	109.0
N5—Mn3—N6	89.20 (13)	N3—C6—H6B	109.0
N7—Mn3—N6	83.44 (12)	C7—C6—H6B	109.0
N8—Mn3—N6	95.81 (12)	H6A—C6—H6B	107.8
O8—Mn3—Mn4	43.30 (8)	C8—C7—C6	114.8 (4)
O7—Mn3—Mn4	43.48 (8)	C8—C7—H7A	108.6
N5—Mn3—Mn4	99.53 (9)	C6—C7—H7A	108.6
N7—Mn3—Mn4	91.41 (9)	C8—C7—H7B	108.6
N8—Mn3—Mn4	132.13 (9)	C6—C7—H7B	108.6
N6—Mn3—Mn4	131.84 (9)	H7A—C7—H7B	107.5
O8—Mn4—O7	81.51 (11)	N4—C8—C7	112.9 (4)
O8—Mn4—O11	172.17 (11)	N4—C8—H8A	109.0
O7—Mn4—O11	92.09 (11)	C7—C8—H8A	109.0
O8—Mn4—O5^i^	93.45 (11)	N4—C8—H8B	109.0
O7—Mn4—O5^i^	172.62 (11)	C7—C8—H8B	109.0
O11—Mn4—O5^i^	93.34 (10)	H8A—C8—H8B	107.8
O8—Mn4—O9	92.53 (10)	C10—C9—N4	107.4 (4)
O7—Mn4—O9	99.40 (10)	C10—C9—H9A	110.2
O11—Mn4—O9	84.01 (10)	N4—C9—H9A	110.2
O5^i^—Mn4—O9	86.15 (10)	C10—C9—H9B	110.2
O8—Mn4—O10	95.15 (10)	N4—C9—H9B	110.2
O7—Mn4—O10	88.53 (10)	H9A—C9—H9B	108.5
O11—Mn4—O10	89.17 (10)	N1—C10—C9	107.5 (4)
O5^i^—Mn4—O10	86.55 (10)	N1—C10—H10A	110.2
O9—Mn4—O10	169.69 (9)	C9—C10—H10A	110.2
O8—Mn4—Mn3	40.60 (8)	N1—C10—H10B	110.2
O7—Mn4—Mn3	41.04 (8)	C9—C10—H10B	110.2
O11—Mn4—Mn3	133.13 (8)	H10A—C10—H10B	108.5
O5^i^—Mn4—Mn3	133.36 (8)	N5—C11—C12	113.8 (3)
O9—Mn4—Mn3	100.44 (7)	N5—C11—H11A	108.8
O10—Mn4—Mn3	89.86 (6)	C12—C11—H11A	108.8
O15—Mn5—O14	86.53 (11)	N5—C11—H11B	108.8
O15—Mn5—N11	92.78 (14)	C12—C11—H11B	108.8
O14—Mn5—N11	94.52 (14)	H11A—C11—H11B	107.7
O15—Mn5—N9	94.10 (13)	C11—C12—C13	114.0 (3)
O14—Mn5—N9	93.53 (13)	C11—C12—H12A	108.7
N11—Mn5—N9	169.71 (15)	C13—C12—H12A	108.7
O15—Mn5—N10	89.18 (13)	C11—C12—H12B	108.7
O14—Mn5—N10	175.15 (13)	C13—C12—H12B	108.7
N11—Mn5—N10	83.40 (14)	H12A—C12—H12B	107.6
N9—Mn5—N10	89.04 (14)	N6—C13—C12	113.4 (3)
O15—Mn5—N12	175.04 (14)	N6—C13—H13A	108.9
O14—Mn5—N12	89.28 (14)	C12—C13—H13A	108.9
N11—Mn5—N12	90.20 (17)	N6—C13—H13B	108.9
N9—Mn5—N12	83.51 (16)	C12—C13—H13B	108.9
N10—Mn5—N12	95.11 (15)	H13A—C13—H13B	107.7
O15—Mn5—Mn6	43.54 (8)	N6—C14—C15	108.3 (3)
O14—Mn5—Mn6	43.05 (8)	N6—C14—H14A	110.0
N11—Mn5—Mn6	96.77 (11)	C15—C14—H14A	110.0
N9—Mn5—Mn6	93.48 (10)	N6—C14—H14B	110.0
N10—Mn5—Mn6	132.72 (10)	C15—C14—H14B	110.0
N12—Mn5—Mn6	132.11 (11)	H14A—C14—H14B	108.4
O14—Mn6—O15	81.47 (11)	N7—C15—C14	108.6 (3)
O14—Mn6—O11	172.64 (11)	N7—C15—H15A	110.0
O15—Mn6—O11	92.11 (11)	C14—C15—H15A	110.0
O14—Mn6—O6	92.30 (11)	N7—C15—H15B	110.0
O15—Mn6—O6	172.26 (11)	C14—C15—H15B	110.0
O11—Mn6—O6	94.39 (11)	H15A—C15—H15B	108.4
O14—Mn6—O13	89.99 (10)	N7—C16—C17	113.3 (3)
O15—Mn6—O13	93.85 (10)	N7—C16—H16A	108.9
O11—Mn6—O13	86.85 (9)	C17—C16—H16A	108.9
O6—Mn6—O13	90.71 (10)	N7—C16—H16B	108.9
O14—Mn6—O12	97.18 (11)	C17—C16—H16B	108.9
O15—Mn6—O12	93.50 (10)	H16A—C16—H16B	107.7
O11—Mn6—O12	86.74 (10)	C18—C17—C16	114.1 (3)
O6—Mn6—O12	82.68 (10)	C18—C17—H17A	108.7
O13—Mn6—O12	170.42 (10)	C16—C17—H17A	108.7
O14—Mn6—Mn5	40.82 (8)	C18—C17—H17B	108.7
O15—Mn6—Mn5	40.70 (8)	C16—C17—H17B	108.7
O11—Mn6—Mn5	132.53 (8)	H17A—C17—H17B	107.6
O6—Mn6—Mn5	133.08 (8)	N8—C18—C17	112.7 (3)
O13—Mn6—Mn5	90.94 (6)	N8—C18—H18A	109.0
O12—Mn6—Mn5	98.65 (7)	C17—C18—H18A	109.0
Mn1—O1—Mn2	96.45 (12)	N8—C18—H18B	109.0
Mn1—O2—Mn2	96.04 (12)	C17—C18—H18B	109.0
C31—O3—Mn2	126.7 (2)	H18A—C18—H18B	107.8
O13^i^—C31—O3	120.8 (3)	N8—C19—C20	108.2 (3)
O13^i^—C31—O10	120.0 (3)	N8—C19—H19A	110.1
O3—C31—O10	119.2 (3)	C20—C19—H19A	110.1
Mn2—O4—H4D	103 (3)	N8—C19—H19B	110.1
Mn2—O4—H4E	112 (3)	C20—C19—H19B	110.1
H4D—O4—H4E	113 (4)	H19A—C19—H19B	108.4
Mn2—O5—Mn4^i^	141.16 (14)	C19—C20—N5	106.9 (3)
Mn2—O5—H5D	106 (3)	C19—C20—H20A	110.3
Mn4^i^—O5—H5D	106 (3)	N5—C20—H20A	110.3
Mn2—O6—Mn6	140.94 (13)	C19—C20—H20B	110.3
Mn2—O6—H6D	99 (3)	N5—C20—H20B	110.3
Mn6—O6—H6D	111 (3)	H20A—C20—H20B	108.6
Mn3—O7—Mn4	95.48 (12)	N9—C21—C22	113.5 (4)
Mn3—O8—Mn4	96.10 (12)	N9—C21—H21A	108.9
Mn4—O9—H9D	144 (3)	C22—C21—H21A	108.9
Mn4—O9—H9E	110 (3)	N9—C21—H21B	108.9
H9D—O9—H9E	99 (3)	C22—C21—H21B	108.9
C31—O10—Mn4	126.1 (2)	H21A—C21—H21B	107.7
Mn4—O11—Mn6	141.70 (13)	C23—C22—C21	113.9 (3)
Mn4—O11—H11D	110 (3)	C23—C22—H22A	108.8
Mn6—O11—H11D	100 (3)	C21—C22—H22A	108.8
Mn6—O12—H12C	103 (3)	C23—C22—H22B	108.8
Mn6—O12—H12D	121 (3)	C21—C22—H22B	108.8
H12C—O12—H12D	109 (3)	H22A—C22—H22B	107.7
C31^i^—O13—Mn6	125.7 (2)	N10—C23—C22	112.2 (4)
Mn5—O14—Mn6	96.13 (12)	N10—C23—H23A	109.2
Mn5—O15—Mn6	95.76 (12)	C22—C23—H23A	109.2
C10—N1—C1	112.6 (4)	N10—C23—H23B	109.2
C10—N1—Mn1	109.8 (3)	C22—C23—H23B	109.2
C1—N1—Mn1	117.9 (3)	H23A—C23—H23B	107.9
C10—N1—H1	105.1	N10—C24—C25	108.3 (4)
C1—N1—H1	105.1	N10—C24—H24A	110.0
Mn1—N1—H1	105.1	C25—C24—H24A	110.0
C3—N2—C4	109.0 (4)	N10—C24—H24B	110.0
C3—N2—Mn1	116.4 (3)	C25—C24—H24B	110.0
C4—N2—Mn1	105.0 (3)	H24A—C24—H24B	108.4
C3—N2—H2	108.7	C24—C25—N11	107.2 (4)
C4—N2—H2	108.7	C24—C25—H25A	110.3
Mn1—N2—H2	108.7	N11—C25—H25A	110.3
C5—N3—C6	113.7 (3)	C24—C25—H25B	110.3
C5—N3—Mn1	109.6 (3)	N11—C25—H25B	110.3
C6—N3—Mn1	117.2 (3)	H25A—C25—H25B	108.5
C5—N3—H3	105.0	N11—C26—C27	113.2 (4)
C6—N3—H3	105.0	N11—C26—H26A	108.9
Mn1—N3—H3	105.0	C27—C26—H26A	108.9
C8—N4—C9	110.4 (4)	N11—C26—H26B	108.9
C8—N4—Mn1	114.8 (3)	C27—C26—H26B	108.9
C9—N4—Mn1	105.9 (3)	H26A—C26—H26B	107.8
C8—N4—H4	108.5	C26—C27—C28	114.8 (5)
C9—N4—H4	108.5	C26—C27—H27A	108.6
Mn1—N4—H4	108.5	C28—C27—H27A	108.6
C11—N5—C20	112.7 (3)	C26—C27—H27B	108.6
C11—N5—Mn3	117.3 (2)	C28—C27—H27B	108.6
C20—N5—Mn3	110.1 (2)	H27A—C27—H27B	107.6
C11—N5—H5	105.2	N12—C28—C27	112.5 (5)
C20—N5—H5	105.2	N12—C28—H28A	109.1
Mn3—N5—H5	105.2	C27—C28—H28A	109.1
C13—N6—C14	110.1 (3)	N12—C28—H28B	109.1
C13—N6—Mn3	116.1 (2)	C27—C28—H28B	109.1
C14—N6—Mn3	105.7 (2)	H28A—C28—H28B	107.8
C13—N6—H6	108.2	N12—C29—C30	109.0 (4)
C14—N6—H6	108.2	N12—C29—H29A	109.9
Mn3—N6—H6	108.2	C30—C29—H29A	109.9
C16—N7—C15	113.8 (3)	N12—C29—H29B	109.9
C16—N7—Mn3	117.5 (2)	C30—C29—H29B	109.9
C15—N7—Mn3	110.0 (2)	H29A—C29—H29B	108.3
C16—N7—H7	104.7	N9—C30—C29	108.1 (4)
C15—N7—H7	104.7	N9—C30—H30A	110.1
Mn3—N7—H7	104.7	C29—C30—H30A	110.1
C18—N8—C19	110.7 (3)	N9—C30—H30B	110.1
C18—N8—Mn3	116.4 (2)	C29—C30—H30B	110.1
C19—N8—Mn3	106.3 (2)	H30A—C30—H30B	108.4
C18—N8—H8	107.7	O27—Cl3—O28	116.6 (4)
C19—N8—H8	107.7	O29—Cl4—O26A	120.7 (8)
Mn3—N8—H8	107.7	O30—Cl4—O26A	94.1 (7)
C30—N9—C21	113.1 (3)	O31^ii^—Cl5A—O20	125.5 (7)
C30—N9—Mn5	110.0 (3)	Cl5B—O20—Cl5A	144.3 (10)
C21—N9—Mn5	118.0 (3)		
			
O2—Mn1—Mn2—O1	−174.57 (19)	N3—Mn1—N1—C10	37.6 (10)
N3—Mn1—Mn2—O1	93.85 (16)	N2—Mn1—N1—C10	82.9 (4)
N1—Mn1—Mn2—O1	−85.59 (17)	N4—Mn1—N1—C10	−11.6 (4)
N2—Mn1—Mn2—O1	10.20 (19)	Mn2—Mn1—N1—C10	−145.4 (3)
N4—Mn1—Mn2—O1	−174.5 (2)	O2—Mn1—N1—C1	127.8 (3)
O1—Mn1—Mn2—O2	174.57 (19)	O1—Mn1—N1—C1	41.2 (3)
N3—Mn1—Mn2—O2	−91.58 (17)	N3—Mn1—N1—C1	−93.0 (8)
N1—Mn1—Mn2—O2	88.98 (18)	N2—Mn1—N1—C1	−47.8 (3)
N2—Mn1—Mn2—O2	−175.2 (2)	N4—Mn1—N1—C1	−142.3 (3)
N4—Mn1—Mn2—O2	0.0 (2)	Mn2—Mn1—N1—C1	83.9 (3)
O2—Mn1—Mn2—O5	−172.38 (17)	O1—Mn1—N2—C3	−46.7 (3)
O1—Mn1—Mn2—O5	2.18 (16)	N3—Mn1—N2—C3	−141.0 (3)
N3—Mn1—Mn2—O5	96.03 (15)	N1—Mn1—N2—C3	46.6 (3)
N1—Mn1—Mn2—O5	−83.40 (15)	N4—Mn1—N2—C3	129.7 (3)
N2—Mn1—Mn2—O5	12.38 (18)	Mn2—Mn1—N2—C3	−53.7 (4)
N4—Mn1—Mn2—O5	−172.35 (18)	O1—Mn1—N2—C4	73.9 (3)
O2—Mn1—Mn2—O6	14.10 (17)	N3—Mn1—N2—C4	−20.4 (3)
O1—Mn1—Mn2—O6	−171.34 (17)	N1—Mn1—N2—C4	167.2 (3)
N3—Mn1—Mn2—O6	−77.49 (15)	N4—Mn1—N2—C4	−109.6 (3)
N1—Mn1—Mn2—O6	103.07 (16)	Mn2—Mn1—N2—C4	67.0 (3)
N2—Mn1—Mn2—O6	−161.14 (18)	O2—Mn1—N3—C5	176.5 (3)
N4—Mn1—Mn2—O6	14.1 (2)	O1—Mn1—N3—C5	−97.1 (3)
O2—Mn1—Mn2—O3	98.13 (15)	N1—Mn1—N3—C5	37.1 (9)
O1—Mn1—Mn2—O3	−87.30 (14)	N2—Mn1—N3—C5	−8.4 (3)
N3—Mn1—Mn2—O3	6.55 (12)	N4—Mn1—N3—C5	85.9 (3)
N1—Mn1—Mn2—O3	−172.89 (13)	Mn2—Mn1—N3—C5	−139.9 (3)
N2—Mn1—Mn2—O3	−77.10 (16)	O2—Mn1—N3—C6	45.0 (3)
N4—Mn1—Mn2—O3	98.17 (17)	O1—Mn1—N3—C6	131.4 (3)
O2—Mn1—Mn2—O4	−80.04 (16)	N1—Mn1—N3—C6	−94.4 (8)
O1—Mn1—Mn2—O4	94.53 (15)	N2—Mn1—N3—C6	−140.0 (3)
N3—Mn1—Mn2—O4	−171.62 (12)	N4—Mn1—N3—C6	−45.7 (3)
N1—Mn1—Mn2—O4	8.94 (13)	Mn2—Mn1—N3—C6	88.6 (3)
N2—Mn1—Mn2—O4	104.73 (16)	O2—Mn1—N4—C8	−44.9 (3)
N4—Mn1—Mn2—O4	−80.00 (17)	N3—Mn1—N4—C8	47.7 (3)
O7—Mn3—Mn4—O8	173.98 (17)	N1—Mn1—N4—C8	−140.3 (4)
N5—Mn3—Mn4—O8	87.63 (15)	N2—Mn1—N4—C8	131.6 (3)
N7—Mn3—Mn4—O8	−92.38 (15)	Mn2—Mn1—N4—C8	−44.9 (4)
N8—Mn3—Mn4—O8	−1.59 (17)	O2—Mn1—N4—C9	77.2 (3)
N6—Mn3—Mn4—O8	−174.83 (17)	N3—Mn1—N4—C9	169.8 (3)
O8—Mn3—Mn4—O7	−173.98 (17)	N1—Mn1—N4—C9	−18.3 (3)
N5—Mn3—Mn4—O7	−86.36 (15)	N2—Mn1—N4—C9	−106.4 (3)
N7—Mn3—Mn4—O7	93.64 (15)	Mn2—Mn1—N4—C9	77.2 (3)
N8—Mn3—Mn4—O7	−175.57 (17)	O8—Mn3—N5—C11	130.4 (3)
N6—Mn3—Mn4—O7	11.19 (16)	O7—Mn3—N5—C11	43.4 (3)
O8—Mn3—Mn4—O11	−173.18 (16)	N7—Mn3—N5—C11	−93.1 (7)
O7—Mn3—Mn4—O11	0.81 (15)	N8—Mn3—N5—C11	−141.4 (3)
N5—Mn3—Mn4—O11	−85.55 (14)	N6—Mn3—N5—C11	−45.5 (3)
N7—Mn3—Mn4—O11	94.45 (14)	Mn4—Mn3—N5—C11	86.9 (3)
N8—Mn3—Mn4—O11	−174.76 (16)	O8—Mn3—N5—C20	−99.0 (3)
N6—Mn3—Mn4—O11	12.00 (16)	O7—Mn3—N5—C20	174.0 (3)
O8—Mn3—Mn4—O5^i^	12.97 (15)	N7—Mn3—N5—C20	37.6 (8)
O7—Mn3—Mn4—O5^i^	−173.04 (16)	N8—Mn3—N5—C20	−10.8 (3)
N5—Mn3—Mn4—O5^i^	100.60 (14)	N6—Mn3—N5—C20	85.2 (3)
N7—Mn3—Mn4—O5^i^	−79.40 (14)	Mn4—Mn3—N5—C20	−142.5 (2)
N8—Mn3—Mn4—O5^i^	11.39 (17)	O7—Mn3—N6—C13	−48.7 (3)
N6—Mn3—Mn4—O5^i^	−161.85 (15)	N5—Mn3—N6—C13	45.7 (3)
O8—Mn3—Mn4—O9	−81.61 (14)	N7—Mn3—N6—C13	−142.4 (3)
O7—Mn3—Mn4—O9	92.37 (14)	N8—Mn3—N6—C13	128.6 (3)
N5—Mn3—Mn4—O9	6.01 (12)	Mn4—Mn3—N6—C13	−56.4 (3)
N7—Mn3—Mn4—O9	−173.99 (12)	O7—Mn3—N6—C14	73.6 (2)
N8—Mn3—Mn4—O9	−83.20 (14)	N5—Mn3—N6—C14	168.1 (2)
N6—Mn3—Mn4—O9	103.56 (14)	N7—Mn3—N6—C14	−20.1 (2)
O8—Mn3—Mn4—O10	98.10 (13)	N8—Mn3—N6—C14	−109.0 (2)
O7—Mn3—Mn4—O10	−87.92 (13)	Mn4—Mn3—N6—C14	65.9 (3)
N5—Mn3—Mn4—O10	−174.28 (11)	O8—Mn3—N7—C16	44.0 (3)
N7—Mn3—Mn4—O10	5.72 (11)	O7—Mn3—N7—C16	130.8 (2)
N8—Mn3—Mn4—O10	96.51 (14)	N5—Mn3—N7—C16	−92.7 (7)
N6—Mn3—Mn4—O10	−76.73 (13)	N8—Mn3—N7—C16	−44.8 (3)
O15—Mn5—Mn6—O14	176.28 (17)	N6—Mn3—N7—C16	−140.7 (3)
N11—Mn5—Mn6—O14	89.38 (17)	Mn4—Mn3—N7—C16	87.4 (2)
N9—Mn5—Mn6—O14	−91.44 (16)	O8—Mn3—N7—C15	176.5 (2)
N10—Mn5—Mn6—O14	176.65 (18)	O7—Mn3—N7—C15	−96.7 (2)
N12—Mn5—Mn6—O14	−7.0 (2)	N5—Mn3—N7—C15	39.8 (8)
O14—Mn5—Mn6—O15	−176.28 (17)	N8—Mn3—N7—C15	87.7 (2)
N11—Mn5—Mn6—O15	−86.89 (17)	N6—Mn3—N7—C15	−8.2 (2)
N9—Mn5—Mn6—O15	92.28 (16)	Mn4—Mn3—N7—C15	−140.2 (2)
N10—Mn5—Mn6—O15	0.37 (17)	O8—Mn3—N8—C18	−47.0 (3)
N12—Mn5—Mn6—O15	176.7 (2)	N5—Mn3—N8—C18	−142.5 (3)
O15—Mn5—Mn6—O11	−8.24 (15)	N7—Mn3—N8—C18	45.7 (3)
O14—Mn5—Mn6—O11	175.48 (16)	N6—Mn3—N8—C18	129.0 (3)
N11—Mn5—Mn6—O11	−95.13 (16)	Mn4—Mn3—N8—C18	−45.9 (3)
N9—Mn5—Mn6—O11	84.04 (14)	N5—Mn3—N8—C19	−18.7 (2)
N10—Mn5—Mn6—O11	−7.87 (17)	N7—Mn3—N8—C19	169.4 (3)
N12—Mn5—Mn6—O11	168.44 (18)	N6—Mn3—N8—C19	−107.2 (2)
O15—Mn5—Mn6—O6	173.33 (16)	Mn4—Mn3—N8—C19	77.9 (3)
O14—Mn5—Mn6—O6	−2.95 (16)	O15—Mn5—N9—C30	−175.0 (3)
N11—Mn5—Mn6—O6	86.44 (16)	O14—Mn5—N9—C30	98.2 (3)
N9—Mn5—Mn6—O6	−94.39 (15)	N11—Mn5—N9—C30	−43.2 (10)
N10—Mn5—Mn6—O6	173.70 (16)	N10—Mn5—N9—C30	−85.9 (3)
N12—Mn5—Mn6—O6	−10.0 (2)	N12—Mn5—N9—C30	9.4 (3)
O15—Mn5—Mn6—O13	−94.82 (13)	Mn6—Mn5—N9—C30	141.4 (3)
O14—Mn5—Mn6—O13	88.90 (14)	O15—Mn5—N9—C21	−43.2 (3)
N11—Mn5—Mn6—O13	178.29 (13)	O14—Mn5—N9—C21	−130.0 (3)
N9—Mn5—Mn6—O13	−2.54 (12)	N11—Mn5—N9—C21	88.6 (9)
N10—Mn5—Mn6—O13	−94.45 (15)	N10—Mn5—N9—C21	45.9 (3)
N12—Mn5—Mn6—O13	81.86 (17)	N12—Mn5—N9—C21	141.1 (3)
O15—Mn5—Mn6—O12	85.29 (14)	Mn6—Mn5—N9—C21	−86.9 (3)
O14—Mn5—Mn6—O12	−90.99 (14)	O15—Mn5—N10—C24	−74.8 (3)
N11—Mn5—Mn6—O12	−1.60 (14)	N11—Mn5—N10—C24	18.1 (3)
N9—Mn5—Mn6—O12	177.57 (12)	N9—Mn5—N10—C24	−168.9 (3)
N10—Mn5—Mn6—O12	85.66 (15)	N12—Mn5—N10—C24	107.7 (3)
N12—Mn5—Mn6—O12	−98.03 (17)	Mn6—Mn5—N10—C24	−75.0 (3)
O2—Mn1—O1—Mn2	3.75 (13)	O15—Mn5—N10—C23	46.4 (3)
N3—Mn1—O1—Mn2	−88.59 (14)	N11—Mn5—N10—C23	139.3 (3)
N1—Mn1—O1—Mn2	99.07 (15)	N9—Mn5—N10—C23	−47.7 (3)
N2—Mn1—O1—Mn2	−172.37 (14)	N12—Mn5—N10—C23	−131.1 (3)
O2—Mn2—O1—Mn1	−3.56 (12)	Mn6—Mn5—N10—C23	46.1 (3)
O5—Mn2—O1—Mn1	−178.38 (12)	O15—Mn5—N11—C26	−129.2 (3)
O3—Mn2—O1—Mn1	91.53 (11)	O14—Mn5—N11—C26	−42.4 (3)
O4—Mn2—O1—Mn1	−94.63 (12)	N9—Mn5—N11—C26	98.9 (9)
O1—Mn1—O2—Mn2	−3.70 (13)	N10—Mn5—N11—C26	142.0 (4)
N3—Mn1—O2—Mn2	90.28 (14)	N12—Mn5—N11—C26	46.8 (3)
N1—Mn1—O2—Mn2	−96.68 (14)	Mn6—Mn5—N11—C26	−85.7 (3)
N4—Mn1—O2—Mn2	−179.97 (15)	O15—Mn5—N11—C25	100.1 (4)
O1—Mn2—O2—Mn1	3.59 (12)	O14—Mn5—N11—C25	−173.1 (3)
O6—Mn2—O2—Mn1	−169.83 (12)	N9—Mn5—N11—C25	−31.7 (11)
O3—Mn2—O2—Mn1	−83.28 (12)	N10—Mn5—N11—C25	11.3 (4)
O4—Mn2—O2—Mn1	105.40 (13)	N12—Mn5—N11—C25	−83.8 (4)
O1—Mn2—O3—C31	178.5 (3)	Mn6—Mn5—N11—C25	143.7 (3)
O2—Mn2—O3—C31	−100.7 (3)	O14—Mn5—N12—C29	−74.7 (3)
O5—Mn2—O3—C31	87.0 (3)	N11—Mn5—N12—C29	−169.2 (3)
O6—Mn2—O3—C31	−6.9 (3)	N9—Mn5—N12—C29	18.9 (3)
O4—Mn2—O3—C31	30.2 (6)	N10—Mn5—N12—C29	107.4 (3)
Mn1—Mn2—O3—C31	−140.8 (3)	Mn6—Mn5—N12—C29	−69.9 (3)
Mn2—O3—C31—O13^i^	−141.3 (3)	N11—Mn5—N12—C28	−46.4 (4)
Mn2—O3—C31—O10	38.1 (4)	N9—Mn5—N12—C28	141.7 (4)
O1—Mn2—O5—Mn4^i^	−48.7 (2)	N10—Mn5—N12—C28	−129.8 (4)
O6—Mn2—O5—Mn4^i^	125.2 (2)	Mn6—Mn5—N12—C28	52.9 (4)
O3—Mn2—O5—Mn4^i^	38.9 (2)	C10—N1—C1—C2	−68.1 (5)
O4—Mn2—O5—Mn4^i^	−150.8 (2)	Mn1—N1—C1—C2	61.3 (5)
Mn1—Mn2—O5—Mn4^i^	−50.2 (3)	N1—C1—C2—C3	−64.4 (6)
O2—Mn2—O6—Mn6	−143.2 (2)	C1—C2—C3—N2	65.0 (6)
O5—Mn2—O6—Mn6	32.5 (2)	C4—N2—C3—C2	−178.6 (4)
O3—Mn2—O6—Mn6	122.4 (2)	Mn1—N2—C3—C2	−60.1 (5)
O4—Mn2—O6—Mn6	−50.7 (2)	C3—N2—C4—C5	171.2 (3)
Mn1—Mn2—O6—Mn6	−152.32 (16)	Mn1—N2—C4—C5	45.8 (4)
O14—Mn6—O6—Mn2	54.3 (2)	C6—N3—C5—C4	169.5 (4)
O11—Mn6—O6—Mn2	−122.6 (2)	Mn1—N3—C5—C4	36.2 (4)
O13—Mn6—O6—Mn2	−35.7 (2)	N2—C4—C5—N3	−55.4 (5)
O12—Mn6—O6—Mn2	151.3 (2)	C5—N3—C6—C7	−71.2 (6)
Mn5—Mn6—O6—Mn2	56.2 (3)	Mn1—N3—C6—C7	58.4 (5)
O8—Mn3—O7—Mn4	4.13 (11)	N3—C6—C7—C8	−64.0 (6)
N5—Mn3—O7—Mn4	99.20 (13)	C9—N4—C8—C7	176.7 (4)
N7—Mn3—O7—Mn4	−88.33 (12)	Mn1—N4—C8—C7	−63.7 (4)
N6—Mn3—O7—Mn4	−171.69 (12)	C6—C7—C8—N4	67.9 (5)
O8—Mn4—O7—Mn3	−3.95 (11)	C8—N4—C9—C10	169.7 (4)
O11—Mn4—O7—Mn3	−179.41 (11)	Mn1—N4—C9—C10	44.9 (4)
O9—Mn4—O7—Mn3	−95.13 (11)	C1—N1—C10—C9	173.2 (4)
O10—Mn4—O7—Mn3	91.48 (11)	Mn1—N1—C10—C9	39.8 (5)
O7—Mn3—O8—Mn4	−4.14 (11)	N4—C9—C10—N1	−56.6 (5)
N5—Mn3—O8—Mn4	−98.28 (13)	C20—N5—C11—C12	−68.6 (4)
N7—Mn3—O8—Mn4	89.24 (12)	Mn3—N5—C11—C12	60.9 (4)
N8—Mn3—O8—Mn4	178.82 (12)	N5—C11—C12—C13	−65.1 (5)
O7—Mn4—O8—Mn3	3.99 (11)	C14—N6—C13—C12	179.2 (3)
O5^i^—Mn4—O8—Mn3	−170.59 (11)	Mn3—N6—C13—C12	−60.8 (4)
O9—Mn4—O8—Mn3	103.12 (11)	C11—C12—C13—N6	65.8 (4)
O10—Mn4—O8—Mn3	−83.75 (11)	C13—N6—C14—C15	171.1 (3)
O13^i^—C31—O10—Mn4	36.4 (4)	Mn3—N6—C14—C15	44.9 (3)
O3—C31—O10—Mn4	−142.9 (2)	C16—N7—C15—C14	169.8 (3)
O8—Mn4—O10—C31	−97.8 (3)	Mn3—N7—C15—C14	35.5 (4)
O7—Mn4—O10—C31	−179.2 (3)	N6—C14—C15—N7	−54.1 (4)
O11—Mn4—O10—C31	88.7 (3)	C15—N7—C16—C17	−71.1 (4)
O5^i^—Mn4—O10—C31	−4.7 (3)	Mn3—N7—C16—C17	59.6 (4)
O9—Mn4—O10—C31	40.3 (7)	N7—C16—C17—C18	−65.4 (5)
Mn3—Mn4—O10—C31	−138.1 (3)	C19—N8—C18—C17	177.0 (3)
O7—Mn4—O11—Mn6	−49.4 (2)	Mn3—N8—C18—C17	−61.6 (4)
O5^i^—Mn4—O11—Mn6	125.6 (2)	C16—C17—C18—N8	66.8 (4)
O9—Mn4—O11—Mn6	−148.7 (2)	C18—N8—C19—C20	172.5 (3)
O10—Mn4—O11—Mn6	39.1 (2)	Mn3—N8—C19—C20	45.2 (3)
Mn3—Mn4—O11—Mn6	−50.0 (3)	N8—C19—C20—N5	−55.6 (4)
O15—Mn6—O11—Mn4	140.5 (2)	C11—N5—C20—C19	171.3 (3)
O6—Mn6—O11—Mn4	−35.3 (2)	Mn3—N5—C20—C19	38.2 (4)
O13—Mn6—O11—Mn4	−125.7 (2)	C30—N9—C21—C22	71.0 (5)
O12—Mn6—O11—Mn4	47.1 (2)	Mn5—N9—C21—C22	−59.4 (5)
Mn5—Mn6—O11—Mn4	145.89 (16)	N9—C21—C22—C23	64.0 (6)
O14—Mn6—O13—C31^i^	178.3 (3)	C24—N10—C23—C22	−176.9 (3)
O15—Mn6—O13—C31^i^	96.8 (3)	Mn5—N10—C23—C22	63.5 (4)
O11—Mn6—O13—C31^i^	4.9 (3)	C21—C22—C23—N10	−66.7 (5)
O6—Mn6—O13—C31^i^	−89.4 (3)	C23—N10—C24—C25	−170.3 (4)
Mn5—Mn6—O13—C31^i^	137.5 (3)	Mn5—N10—C24—C25	−44.8 (4)
O15—Mn5—O14—Mn6	−2.57 (12)	N10—C24—C25—N11	56.0 (5)
N11—Mn5—O14—Mn6	−95.08 (15)	C26—N11—C25—C24	−172.1 (4)
N9—Mn5—O14—Mn6	91.33 (14)	Mn5—N11—C25—C24	−38.8 (5)
N12—Mn5—O14—Mn6	174.78 (15)	C25—N11—C26—C27	68.5 (6)
O15—Mn6—O14—Mn5	2.45 (11)	Mn5—N11—C26—C27	−60.6 (6)
O6—Mn6—O14—Mn5	177.85 (11)	N11—C26—C27—C28	65.0 (7)
O13—Mn6—O14—Mn5	−91.44 (11)	C29—N12—C28—C27	−178.9 (4)
O12—Mn6—O14—Mn5	94.94 (12)	Mn5—N12—C28—C27	61.0 (5)
O14—Mn5—O15—Mn6	2.54 (12)	C26—C27—C28—N12	−66.4 (6)
N11—Mn5—O15—Mn6	96.91 (14)	C28—N12—C29—C30	−168.7 (4)
N9—Mn5—O15—Mn6	−90.75 (13)	Mn5—N12—C29—C30	−43.7 (4)
N10—Mn5—O15—Mn6	−179.73 (13)	C21—N9—C30—C29	−169.8 (4)
O14—Mn6—O15—Mn5	−2.46 (11)	Mn5—N9—C30—C29	−35.6 (5)
O11—Mn6—O15—Mn5	173.93 (11)	N12—C29—C30—N9	53.5 (5)
O13—Mn6—O15—Mn5	86.95 (11)	O31^ii^—Cl5A—O20—Cl5B	162.0 (15)
O12—Mn6—O15—Mn5	−99.20 (11)	O26A—Cl4—O29—O30	14 (2)
O2—Mn1—N1—C10	−101.6 (4)	O26A—Cl4—O30—O29	−168 (2)
O1—Mn1—N1—C10	171.9 (4)		

Symmetry codes: (i) −*x*+1, −*y*+1, −*z*+1; (ii) *x*−1, *y*, *z*; (iii) *x*+1, *y*, *z*.

### Hydrogen-bond geometry (Å, °)

**Table:**

*D*—H···*A*	*D*—H	H···*A*	*D*···*A*	*D*—H···*A*
N6—H6···Cl1	0.93	2.51	3.413 (3)	163
N8—H8···Cl1	0.93	2.27	3.198 (3)	174
O4—H4E···Cl2	0.82 (4)	2.33 (3)	3.106 (3)	158 (4)
N1—H1···Cl2	0.93	2.49	3.285 (4)	143
N2—H2···Cl3	0.93	2.46	3.338 (4)	157
N4—H4···Cl3	0.93	2.46	3.384 (4)	174
N11—H11···Cl4	0.93	2.37	3.148 (5)	141
N10—H10···Cl5A	0.93	2.68	3.523 (6)	152
N10—H10···Cl5B	0.93	2.49	3.185 (12)	132
N5—H5···Cl6	0.93	2.32	3.138 (4)	147
O4—H4D···O14	0.82 (4)	1.90 (2)	2.701 (4)	166 (4)
O5—H5D···O13	0.84 (4)	1.86 (2)	2.674 (3)	164 (4)
O6—H6D···O10	0.82 (4)	1.88 (2)	2.681 (4)	166 (4)
O9—H9E···O1^i^	0.87 (3)	1.83 (2)	2.665 (4)	161 (4)
O11—H11D···O3^i^	0.83 (2)	1.85 (2)	2.675 (3)	172 (4)
O12—H12C···O7	0.83 (5)	1.88 (5)	2.704 (4)	171 (4)
O12—H12D···Cl4	0.82 (2)	2.26 (2)	3.065 (4)	165 (4)
N2—H2···O28	0.93	2.08	2.952 (9)	156
N3—H3···O3	0.93	1.99	2.798 (4)	144
N7—H7···O10	0.93	1.99	2.790 (4)	143
N9—H9···O13	0.93	2.14	2.896 (4)	138
N10—H10···O20	0.93	2.02	2.912 (10)	160

Symmetry codes: (i) −*x*+1, −*y*+1, −*z*+1.
